# Skin Deep: Uncovering the Early Events of Crimean–Congo Hemorrhagic Fever Virus at the Tick–Host–Virus Interface

**DOI:** 10.3390/v18040429

**Published:** 2026-04-01

**Authors:** Catherine Olal, Megan Burch, Dennis Bente

**Affiliations:** 1 Department of Microbiology and Immunology, University of Texas Medical Branch, Galveston, TX 77550, USA; 2 Galveston National Laboratory, University of Texas Medical Branch, Galveston, TX 77550, USA

**Keywords:** CCHFV, tick, saliva, skin, cutaneous immunity, arbovirus

## Abstract

Crimean-Congo hemorrhagic fever virus (CCHFV) is transmitted predominantly through the bite of infected *Hyalomma* ticks, yet the earliest events at the vector–host–virus interface in human skin remain largely undefined. This review synthesizes current knowledge of human cutaneous structure and immunity, tick feeding biology, and salivary immunomodulation to propose how local skin responses may shape systemic outcomes of CCHFV disease. We detail the roles and permissiveness of major skin-resident and infiltrating cell types, including keratinocytes, melanocytes, Langerhans cells, dermal dendritic cells, monocytes/macrophages, fibroblasts, granulocytes, T cells, B cells, NK cells, and innate lymphoid cells, in antiviral defense and as potential early targets or carriers of CCHFV. Emphasis is placed on how tick saliva components reprogram the cutaneous microenvironment, alter interferon, complement, inflammasome, and cytokine pathways, and may enable saliva-assisted transmission and viral dissemination from the dermis. We highlight mounting evidence from other arboviruses demonstrating that the skin can act as both a barrier and a major amplifying organ, and we extrapolate testable hypotheses on how early cutaneous immune dynamics might influence CCHFV severity and hemorrhagic manifestations. Finally, we outline key knowledge gaps that, if answered, may inform the development of vaccines and therapeutics that harness cutaneous immunity to block systemic spread.

## 1. Introduction

The skin, the largest organ in the body, protects us from environmental, chemical, and physical hazards and pathogens. It is an active immune organ composed of a plethora of cell types, including innate and adaptive immune cells that work in concert to halt the replication and systemic spread of pathogens and prevent disease [1]. The structure of the skin is integral to its barrier function and limits pathogen entry. The skin also consists of a complex ecosystem of microorganisms, including commensal bacteria, mites, fungi, and viruses, that contribute to its health by maintaining homeostasis and bolstering its protective function and immune competence [2].

Disease vectors such as mosquitoes and ticks can circumvent the skin’s barrier function by depositing the virus directly into the skin while feeding, increasing the probability of infection in susceptible individuals. Ticks effectively burrow into the host’s skin and directly deposit pathogens, triggering a complex interaction between the tick, host, and pathogen [3].

Despite its expanding range and severe clinical impact, CCHF remains underdiagnosed and underreported in many endemic regions, and no widely licensed, safe, and effective vaccine is yet available for humans. Investigating the pathogenesis of CCHF virus, especially defining viral determinants of virulence and the host factors that influence susceptibility and severe illness, including hemorrhagic manifestations, is critical to inform evidence-based strategies for surveillance, tick and livestock control, infection prevention, and the development of targeted vaccines and therapeutics.

Understanding the vector–host–pathogen interaction remains a crucial element in disease control and can directly contribute to the development of effective countermeasures. Most studies on arthropod-borne transmission of pathogens are performed in animal models such as mice, rats, rabbits, and pigs. Such studies, though very useful, do not fully recapitulate what is observed in humans, in part due to the differences in the structure and cellular composition of human, pig, and mouse skin. 2D and 3D in vitro modelling of human skin using primary cells and skin explants can provide much-needed information on the major cell types involved in the response to infection. Multi-omics approaches can also provide insight into disease pathogenesis. Indeed, the advent of single-cell technologies has led to new insights into immune cell function, the discovery of new cell subsets, and the reclassification of old ones. Incorporating high-dimensional flow cytometry, spatial transcriptomics, and computational approaches such as artificial intelligence and predictive modeling into studies of host cutaneous responses to tick-borne pathogens could greatly improve the spatial, temporal, and mechanistic resolution, enabling more insight into events happening from the tick bite site in the skin to systemically affected organs.

In this review, we discuss the structure and composition of human skin, skin immunity to viral pathogens, and the role of tick feeding and saliva in the early pathogenesis of Crimean-Congo hemorrhagic fever virus (CCHFV). As there is limited information on the cutaneous response to CCHFV, we will include current knowledge on other vector-borne and skin-tropic viruses.

## 2. Crimean-Congo Hemorrhagic Fever Virus—Why Is It Important?

Crimean-Congo hemorrhagic fever (CCHF) is a severe tick-borne viral illness caused by Crimean-Congo hemorrhagic fever virus (CCHFV). CCHFV is a widespread tick-borne pathogen found across Africa, the Middle East, Southeast Asia, and southern and eastern Europe, with its distribution closely mirroring that of its primary vector and reservoir, *Hyalomma* spp. ticks [4]. The virus has a broad host range, infecting multiple animal species, including livestock, but interestingly causes no overt disease in these animals despite measurable viremia and seroconversion [5,6]. The virus, however, can cause severe disease in humans marked by coagulopathy, hemorrhage, and eventually multi-organ failure in the end stages if symptoms are not appropriately managed. CCHFV has broad tropism and infects multiple cell types, including monocytes, dendritic cells (DCs), macrophages (Mφ), endothelial cells, and hepatocytes [7,8,9,10]. The low-density lipoprotein receptor (LDLR), highly expressed by hepatocytes, was recently described as a major cellular entry receptor for CCHFV [11,12,13].

CCHF has a variable mortality rate ranging from 10–50% [14]. It is not yet clear why some individuals remain asymptomatic or only develop mild disease. Currently, little is known about the events that occur at the tick–host–virus interface during and immediately after pathogen transmission. Due to the variability in disease severity, we suspect that the dynamics at the local site of infection can influence the trajectory of illness. It is plausible that the nature and strength of early cutaneous immune responses, such as the rapid recruitment of immune cells, production of antiviral cytokines, and modulation by tick saliva, may help determine whether the virus is contained locally or disseminates systemically. Heterogeneity in clinical outcomes may therefore reflect differences in these initial local immune events, as well as individual variation in skin immunology, the volume and composition of salivary factors delivered, or the identity of the primary infected cell types at the bite site. We hypothesize that early, robust local immune activation might limit viral spread and result in mild disease, whereas delayed or dysregulated cutaneous responses could permit rapid dissemination and more severe outcomes. Investigation into early skin events following tick feeding may thus yield biomarkers predictive of clinical severity and open new avenues for intervention at the earliest stages of infection.

## 3. Tick Saliva—Delivery of the Virus and Modification of the Immune Response

While *Hyalomma* spp. are the primary drivers of transmission, other tick species may also contribute to the maintenance and spread of the virus. Tick bites are thought to be the predominant route of human infection, although transmission can also occur through contact with infected blood or tissues, and in some cases, human-to-human spread [4]. Human cases of CCHFV are often seasonal, coinciding with peak tick activity in spring and summer, and many cases have a history of tick bite or contact, underscoring the role of tick transmission in CCHFV [15,16,17,18,19].

*Hyalomma* spp. ticks, particularly *H. marginatum*, are considered the primary vectors and reservoirs of the CCHFV [4]. *Hyalomma* spp. are generally two- or three-host ticks, with immature stages feeding on small vertebrates and adults primarily feeding on large ungulates, such as livestock [20]. *Hyalomma* spp. that are most commonly found infesting humans are *H. anatolicum*, *H. marginatum*, and *H. aegyptium* [21,22]. These ticks exhibit hunting style host-seeking behavior, actively pursuing hosts, and pose a significant risk of human infestation and potential CCHFV infection.

The tick bite serves as a dynamic immunological interface between the host and vector, with salivary components actively shaping the local immune response. Infection of a host typically occurs through the bite of an infected tick, during which the tick inserts its hypostome and secures its position by cementing its mouthparts to the host. When secured, ticks release a pharmacy of enzymes and proteins through the saliva to facilitate feeding and prevent host immune detection. Arboviruses exploit the feeding behavior and salivary components of the tick vector to facilitate transmission, making tick saliva an important yet understudied component of pathogenesis for CCHFV.

The composition of saliva is unique for each species and dynamic, adjusting throughout the bloodmeal, potentially as an immune evasion mechanism [23]. Tick saliva contains anticoagulants, cytolytics, vasoactive compounds, and other molecules that facilitate blood-feeding and modulate host defenses [24]. These components directly modulate the immune microenvironment of the skin, interfering with the local activation, recruitment, and migration of immune cells, which are key events in early antiviral defense, simultaneously benefiting the tick and viral pathogen. While tick bites are often unrecognized by the host due to the salivary interference of host reaction, tick bites alone can impact a parasitized host. Tick feeding could result in minor complications, such as itching, pain, inflammation, redness, swelling, and potential for secondary infection at the bite site [25], and, albeit rare, major complications, such as paralysis [26].

Pathogens are mostly, if not exclusively, transmitted from ticks to hosts through salivary gland secretions [27]. Host modulation through tick saliva can be exploited to benefit the transmission of pathogens from tick to host, establishing a direct relationship between pathogens and salivary transmission. There is evidence that co-evolution between ticks, vertebrate hosts, and associated pathogens has led to a phenomenon known as saliva-assisted transmission (SAT) [28], in which tick saliva enhances transmission of tick-borne pathogens, either directly or indirectly. Given that CCHFV is likely deposited directly into the dermis by ticks, the interaction between viral particles, salivary components, and resident skin immune cells likely determines whether infection is established or cleared. However, early immunological crosstalk, especially in the context of salivary modulation, remains poorly understood for CCHFV. Variation in salivary composition over the course of feeding, known as sialome switching [29], differences in salivary proteins between species [30], and the volume of saliva secreted [31] may result in a spectrum of infection outcomes. Unraveling the effects of saliva on early CCHFV infection could enhance therapeutic development.

There are no reports of SAT and CCHFV in human infection [32]. While it was observed that *Hyalomma marginatum* saliva can modify the migration of antigen-presenting cells (APC), there was no evidence that the saliva affects CCHFV replication [33]. Early reports on CCHFV indicate that the virus is intimately intertwined with tick saliva, as evidenced by the highest viral titers being found in proliferating tissues, including the salivary glands [34]. However, the timeline to transmission during feeding is unclear. Whether the virus is present in tick salivary secretions during the earliest stages of infection, allowing quick transmission as described for Powassan virus (POWV) [35], or is present in the hemolymph and is then stimulated to enter the salivary glands at the start of feeding, delaying transmission as described with Dugbe virus (DUGV) [36], is of interest.

**Figure 1:**
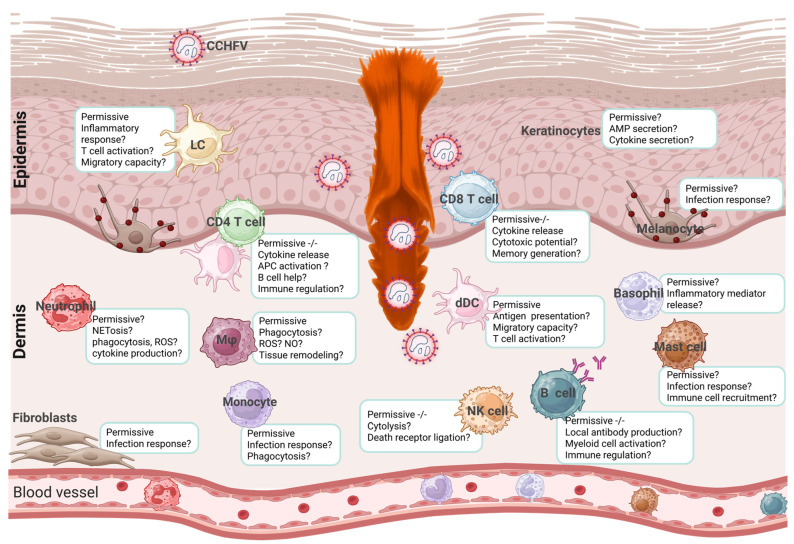
Representation of immune and non-immune cells in the skin, permissiveness to CCHFV, and known and outstanding questions. Created in BioRender. Burch, M. and Olal, C. (2026) https://BioRender.com/nozff6g.

Once CCHFV is transmitted from a tick to the host, it is exposed to a variety of cell types in the skin. The susceptibility and permissiveness of the exposed cell types determine the trajectory of infection in the host (Figure 1). The shorter incubation periods of CCHF patients infected by tick bite compared to those infected via exposure to infected blood or tissues suggest that the role of tick saliva in infection outcomes is critical in disease severity [37]. Several salivary proteins have been isolated and investigated for *Hyalomma* spp. (Table 1), shedding light on potential effects on the host, discussed more below. While single components of the salivary gland extract may have shown specific effects, tick saliva works as a symphony, combining and fluctuating to ensure bloodmeal completion. There are likely several counteractions and redundancies in the composition that necessitate a global view to determine its effect on the host and transmitted pathogen. While there are still many unknowns regarding the kinetics and time to delivery of CCHFV from tick to host, saliva is likely to contribute to the early dynamics of infection, potentially affecting downstream pathogenesis and overall severity of illness.

## 4. CCHFV Dermatological Manifestations

CCHFV infection has characteristic dermatological manifestations associated with illness, including petechial rash of the skin that can progress into large cutaneous ecchymoses [4] and a potential butterfly-like rash on the face [56]. These manifestations are driven by systemic inflammatory responses and are not associated with tick saliva. Though the local infection site is often clinically unremarkable, there may be nonspecific reactions, such as a small erythematous papule or mild inflammation, but no characteristic presentations for CCHFV [57]. While there are no obvious changes at the site of infection, dynamic interactions are likely occurring between the tick, virus, and host just below the surface.

## 5. The Skin—From Barrier to Battlefield

Mammalian skin is composed of three main layers. The epidermis, the outermost layer, is directly in contact with the surroundings and is the primary physical barrier and frontline defense against harmful substances in the environment. It consists of keratinocytes, corneocytes, and melanocytes that produce antimicrobial molecules [58,59,60]. Directly beneath is the dermis, which contains various immune cell types and a network of blood and lymphatic vessels. The hypodermis, subjacent to the dermis, is made up of adipose and connective tissue and contains fibroblasts, Mφ, and stromal cells [61,62,63,64].

### 5.1. Structure of the Skin

#### 5.1.1. The Epidermis

The epidermis is made up of four distinct layers: stratum corneum, stratum granulosum, stratum spinosum, and stratum basale. An extra layer, stratum lucidum, is present in the thick skin of the palms of the hands and soles of the feet [65,66]. Melanocytes and keratinocytes are continuously produced by stem cells present in the deepest layer, the stratum basale [66]. They continuously divide and proliferate in the basal layer, after which keratinocytes migrate to the uppermost layer, the stratum granulosum, where they differentiate into corneocytes [67,68]. Immune cells, such as Langerhans cells (LCs), are also present in the epidermis, where they are ideally situated to capture antigens [69]. T cells and dermal dendritic cells (dDCs) circulate within the epidermis and dermis, where they orchestrate the defense against microbes [70].

#### 5.1.2. The Dermis

The dermis forms about 90% of the skin’s total thickness and comprises a network of blood and lymphatic vessels that facilitate the movement of immune cells between the skin, lymph nodes, and the circulatory system [71]. This middle layer is divided into two regions; the papillary dermis, a highly vascularized segment that nourishes and supports the epidermis and contains immune cells; and the reticular region, which is composed of dense irregular tissue and abundant extracellular matrix (ECM) mainly made up of collagen and elastin fibers that contribute to the skin’s elasticity, resilience and provide support [72,73]. In the dermis, a diverse subset of immune cells works in tandem to repel pathogens that overcome the epidermal barrier’s defenses. These cells belong to both the innate and adaptive arms of immunity and, through their interactions, coordinate and potentiate the immune response to invading pathogens. Dermis resident cells include Mφ, DCs, lymphocytes, fibroblasts, natural killer (NK) cells, mast cells, and innate lymphoid cells (ILCs) [62,70,74]. Transient cells such as monocytes, basophils, and neutrophils are present in low numbers in healthy skin [73]. During ongoing infections, these cells migrate into the skin where they mount an immune response.

#### 5.1.3. Hypostome

The depth at which *Hyalomma* ticks penetrate host skin is determined by hypostome length and host epidermal and dermal thickness. In humans, the thickness of the epidermis ranges from 50–100 μm and the dermis between 1000–2000 μm, with greater contrasts being found in thinner regions, such as the eyelids, and thicker regions, such as the palms of the hands. For *Hyalomma* species, the hypostome lengths range from 54–106 μm for larvae [75,76], 156–246 μm for nymphs [75,76,77], 411–442 μm for adult males, and 451–503 μm for adult females [76]. While nymphs and adults may penetrate well into the dermis, larvae may not have sufficient length to reach the dermis in most cases when on a human host. Where the virus may be deposited and at what depth may influence the trajectory of infection based on the composition of host cells in the vicinity.

### 5.2. Cellular Composition

The skin is the first barrier that pathogens must overcome to establish disease. In the event of a failure of the skin’s barrier function and a breach by pathogens, cutaneous innate immune cells respond by releasing inflammatory cues and effector molecules that can curtail further infiltration, replication, and dissemination of the invading microorganisms. Innate immune cells in the skin include phagocytic Mφ, LCs, DCs, inflammatory monocytes, and leukocytes such as basophils, mast cells, eosinophils, and neutrophils [78]. Adaptive immune cells comprise lymphocytes, including different helper T cell subsets, CD8+ T cells, and resident memory T cells (T_RM_).

Innate immune cells express various pattern recognition receptors (PRRs) on their surface, which recognize pathogen-associated molecular patterns (PAMPs) on viruses and bacteria. These PRRs include Toll-like receptors (TLRs), Nod-like receptors (NLRs), Rig-I-like receptors (RLRs), and C-type lectin receptors (CLRs). These receptors recognize conserved structural motifs present in viruses such as single- or double-stranded RNA (ssRNA or dsRNA), viral DNA or proteins, and unmethylated cytosine-guanine motifs (CpG) [79]. Endosomal TLR 3 and 7 and cytosolic RLRs—Melanoderma Differentiation-Associated gene 5 (MDA5) and Retinoic Acid-Inducible gene (RIG-I)—are the main PRRs involved in sensing arboviruses [80,81]. Upon recognizing viral PAMPs, intracellular signaling cascades are initiated that result in the secretion of inflammatory mediators such as cytokines and chemokines, which in turn recruit more immune cells to the site of infection, further activating the surrounding local immune cells and initiating adaptive immunity [82]. In addition to PAMPs, PRRs also recognize damage-associated molecular patterns (DAMPs), molecular structures expressed by apoptotic or senescent host cells [83].

The following sections will describe the cellular composition of the skin and the roles that skin-resident cells play during infection with various pathogens, and how the virus and tick may alter their functions to promote infection.

#### 5.2.1. Keratinocytes

Keratinocytes make up approximately 90% of the cells in the epidermis and contribute to both the barrier and immune functions of the skin. They are the first cells that viruses encounter upon infection. Keratinocytes are permissive to arthropod-borne viruses (arboviruses), including Zika virus (ZIKV), Dengue virus (DENV), and West Nile virus (WNV) [84,85,86].

Keratinocytes produce an array of molecules and lipids with direct and indirect antimicrobial effects after infection. These antimicrobial peptides (AMPs) and fatty acids (AFAs), also produced by cells of the myeloid lineage, have mostly been studied in the context of bacterial infections, where they have been shown to disrupt bacterial cell membranes and/or impede the acquisition of important ions necessary for bacterial growth and survival [59]. The most extensively studied AMPs are cathelicidins, defensins, transferrins, and S100 family proteins. This review will only focus on AMPs produced by cells present in the skin, namely defensins and cathelicidins.

Dead keratinocytes in the stratum granulosum produce defensins—cysteine-rich peptides with immune regulatory activities [87]. Three groups of defensins have been described to date: alpha (α), beta (β), and theta (θ). Only the α and β subgroups have been observed in humans and are produced by keratinocytes, epithelial cells, Mφ, NK cells, and T and B cells. Defensins appear to directly exert their activity against viruses at the entry step or indirectly at different stages of the viral life cycle [88]. Studies have demonstrated the antiviral activity of defensins against human immunodeficiency virus (HIV), human papillomavirus (HPV), and Herpes simplex virus (HSV), where they function by inhibiting viral replication, preventing virion escape, and preventing viral entry, respectively [89,90,91]. They have also been shown to play a role in wound healing and to modulate the immune response to invading pathogens by recruiting DCs to the sites of infection and inducing their maturation, thus inducing the secretion of proinflammatory cytokines by keratinocytes and Mφ and regulating apoptosis pathways [88].

Human cationic antimicrobial peptide 18 (hCAMP-18—the proprotein) or LL-37—the processed peptide, is the only known cathelicidin produced by humans; its synthesis, together with that of beta-defensin 4, is reliant on the vitamin D antimicrobial pathway [92,93]. In addition to vitamin D, proinflammatory cytokines such as IL-15 can also induce the expression of the human *CAMP* gene, leading to the production of LL-37. However, this process is inconsistent, and infection by various pathogens has been shown to suppress this gene, leading to reduced production of the peptide [93]. On the other hand, anti-inflammatory cytokines such as IL-4, IL-10 and IL-13 are thought to decrease the induction of LL-37 as evidenced by a study that compared the skin biopsies obtained from atopic dermatitis and psoriasis patients. LL-37 expression was significantly reduced in the lesions of atopic dermatitis patients compared to those from psoriatic patients [94].

LL-37 has been shown to remove the outer membrane of enveloped viruses, leading to their destruction. This cathelicidin also functions as a chemoattractant, inducing the migration of immune cells such as neutrophils, monocytes, DCs, and even T cells [95]. The antiviral activity of LL-37 has been demonstrated against HIV, Influenza A virus (IAV), DENV, ZIKV, Vaccinia virus (VACV), and respiratory syncytial virus (RSV) [96,97,98].

It is not yet clear whether CCHFV can replicate in these keratinocytes and what effect their infection has on the overall immune response. Keratinocytes are one of the first cell types exposed to the pathogen and are likely integral in shaping the recruitment response (helping or failing to control spread). The suppression of keratinocyte function by tick saliva may alter the inflammatory response and chemokine signaling, thereby affecting the course and outcome of infection.

#### 5.2.2. Melanocytes

Melanocytes are an integral component of the skin and, together with keratinocytes, contribute to the skin’s barrier function. Melanin is produced by melanocytes, which protects epidermal cells from damaging, DNA-altering UV rays. These cells can regulate the immune response by activating and enhancing cytokine production in endothelial cells, fibroblasts, and T cells. Melanocytes express TLRs, and their ligation activates signaling pathways such as nuclear factor-kB(NF-kB) and Mitogen-activated protein kinase (MAPK), leading to the secretion of cytokines and chemokines, which in turn recruit important immune cells that can aid in limiting viral replication [99]. They also express intercellular adhesion molecule 1 (ICAM-1), CD40, and major histocompatibility complex (MHC) II (in the presence of interferon (IFN)), which are key regulators of T-cell-dependent humoral and cellular immunity. Melanocytes have also been shown to have phagocytic ability and may therefore contribute to pathogen control [99,100].

The synthesis of melanin is a multistep process that begins with the oxidation of the amino acid tyrosine and downstream aromatic compounds [101]. Throughout this process, multiple intermediate compounds with potent antimicrobial activity, including reactive oxygen species (ROS), are produced. The roles of melanocytes and melanin in arboviral infections remain unclear.

### 5.3. Immunological Landscape of the Skin

#### 5.3.1. Langerhans Cells

LCs were first described in 1868 by Paul Langerhans [102] and were classified as APCs by Schuler & Steinman [103]. LCs together with Mφ, DCs, and monocytes constitute the mononuclear phagocyte system (MPS). LCs share ontogeny with Mφ but can migrate from the skin to the lymph nodes after activation, a property they share with DCs [104,105]. They form a network that spans the epidermis, a prime location for sensing and responding to viruses transmitted through the skin. LCs constantly surveil the skin by extending and retracting their dendrites in between keratinocytes, thereby enabling them to sample the surrounding environment [69,106,107]. They are capable of self-renewal and proliferate in the steady state to replenish their numbers. In the presence of inflammatory stimuli such as those produced during a viral infection, the emigrated LCs are replenished by circulating monocytes [105,108,109,110]. LCs express an array of PRRs that they use to sense viral PAMPs. This includes the C-type lectin receptor CD207, also known as langerin, that binds pathogens and is used to identify LCs, though it is also expressed by type 2 conventional DCs (cDC2s) in humans. Upon contact with pathogens, LCs take up antigens via TLRs and CLRs, leading to activation and triggering their migration to the skin-draining lymph nodes (sLNs). During this time, the antigens are processed, and the peptides are loaded onto MHCII molecules for presentation to naive CD4^+^ T cells. LCs can variably induce T helper 1, 2, 17, 22 (T_H1_, T_H2_, T_H17_, and T_H22_) T cell generation depending on the pathogen. Interestingly, LCs can also activate skin-resident memory T cells, which rapidly respond by killing virus-infected cells, highlighting their importance in mediating protection against secondary infections [111].

LCs have been shown to play an immunoregulatory role both in the steady state and under inflammatory conditions. They induce regulatory T cell (T_reg_) formation in the sLNs in the steady state and locally in healthy skin [112]. Studies have shown that antigen targeting to LCs in vivo can lead to cross-tolerance rather than cross-priming, as LCs are less efficient at cross-presenting exogenous antigens compared to DCs [113,114].

HSV infection of LCs induces apoptosis, while LCs appear to have a protective role in HIV infection [115]. In the case of HSV, it is thought that uninfected LCs take up infected apoptotic cells and migrate to the sLN where they transfer the load to cross-presenting DCs that, in turn, activate CD8^+^ T cells [116,117]. A recent study by Helgers et al. [118] demonstrated that both immature and mature LCs are permissive to DENV and readily transmit the virus to DCs. Viral entry is facilitated by Langerin, and LC migration from the epidermis is enhanced, suggesting that these cells may play a role in viral dissemination.

LCs are susceptible and permissive to CCHFV, and infection results in the production of proinflammatory cytokines, specifically IL-6, monocyte chemoattractant protein-1 (MCP-1), and TNF-α [33]. It would be interesting to determine whether CCHFV-infected LCs can transmit the virus to adjacent cells in the skin and to elucidate the underlying molecular mechanisms involved, overall elucidating the role LCs play in viral dissemination.

#### 5.3.2. Dendritic Cells

DCs are often referred to as professional APCs due to their remarkable ability to capture, process, and present antigens to naïve T cells. They are the only cells demonstrated to prime naive T cells and act as a bridge between the innate and adaptive immune systems. They were first described in 1973 by Steinman & Cohn [119], and since then, more studies have revealed the critical role that these cells play in initiating and maintaining host defenses to pathogens. DCs are classified by function and phenotype and can be broadly categorized into 2 groups: plasmacytoid DCs (pDC) and conventional DCs (cDC). cDCs are further divided into type I and type II cDCs based on the expression of phenotypic markers. An additional subset—monocyte-derived DCs (moDCs)—is present in inflammatory conditions [120,121].

cDCs are present in the dermis in the steady state and, like LCs, constantly surveil the skin for invading pathogens. They express TLRs, CLRs, NLRs, and RLRs, which they use to sense and capture viral antigens for presentation to T cells. CLRs and TLRs interact with viral proteins, with the most relevant CLR being DC-SIGN. DC-SIGN can bind and internalize various viruses, including ZIKV, which has been shown to preferentially infect DC-SIGN-expressing DCs over LCs [122]. Mannose receptor (CD206) and Langerin (CD207) are also expressed by dermal DCs [123]. Upon antigen capture, DCs can present antigens locally or migrate to the sLNs, where they initiate and modulate adaptive immunity by activating and driving T cells to release cytokines and other effector molecules that protect the host. One such marker is CCR7, a chemokine receptor that facilitates the migration or homing of immune cells to the draining lymph nodes (dLNs) or secondary lymphoid organs (SLO). DCs upregulate the expression of CCR7, enabling them to respond to CCL19 and CCL21 secreted by lymphatic endothelial cells, facilitating their migration to the dLNs [124,125,126]. DC subsets in the skin comprise CD14^+^CD1a^−^ DCs, CD14^−^CD1a^+^ DCs and CD141^+^ DCs. CD14^−^CD141^+^ DCs are potent CD8^+^ T cell inducers, while CD14^+^ dermal DCs are better at orchestrating humoral immunity. CD14^+^CD141^+^ DCs are thought to play a role in maintaining tissue homeostasis as they produce IL-10, a potent anti-inflammatory cytokine that regulates the immune response by preventing excessive inflammation and tissue damage while ensuring effective pathogen clearance [127,128].

Exogenous antigens, such as viral particles introduced into the skin by ticks during feeding, are processed and presented to CD4^+^ T cells via MHC-II molecules. The priming of T cells by dermal DCs leads to their differentiation into various effector subsets that play distinct roles and contribute to shaping the immune response to the invading pathogen [129]. On the other hand, once DCs have been infected, endogenous antigens derived from replicating viruses are loaded onto MHC-I molecules and presented to CD8+ T cells, which have direct cytotoxic activity and are crucial for defense against viruses [130]. DCs can also redirect exogenous antigens after uptake for loading and presentation via MHC I molecules, a process termed cross-presentation [117,131,132,133]. This unique property makes DCs especially important in initiating and driving immunity to pathogens. As with LCs, dermal DCs recruit and activate skin-resident memory T cells locally by secreting chemokines such as CCL5, CCL18, CCL20, and CCL22 in the event of secondary infection [125].

Dermal DCs are permissive to CCHFV infection and are activated following infection, producing IL-6 and TNF-α, key proinflammatory cytokines that contribute to viral control by activating T cells and inducing the apoptosis of virus-infected cells, respectively [33,134,135].

Tick saliva has been found to modulate DC chemotaxis by downregulating surface receptors involved in the response to CCL3, CCL4, and CCL5 [136]. Additionally, tick saliva has been shown to impair DC function by interfering with differentiation and maturation, processes that are necessary for adequate T cell priming [136]. Tick saliva was also shown to enhance the replication of tick-borne encephalitis virus (TBEV) in DCs by activating the phosphatidylinositol 3-kinase (PI3K)/Akt pathway, thereby reducing apoptosis [137].

pDCs produce copious amounts of type I interferons (IFN-α and IFN-β) in response to viral infection and are key mediators of antiviral immunity. They are present in very low numbers or absent in healthy skin, but during viral infections pDCs infiltrate the skin from the blood [138]. There is accumulating evidence that pDCS mount antiviral responses by sensing virus-infected cells or viral RNA transferred by noninfectious cellular carriers such as exosomes rather than free viruses [139]. pDCs appear less permissive to viruses and rapidly produce type I and III IFNs following viral nucleic acid sensing by endosomal TLR 7 or 9 [139,140]. Type I IFNs create an antiviral state in the immediate surroundings and are especially important to the resolution of viral infections. Although clinical evidence is limited, a few studies have shown that dysregulation of the pDC response is associated with poorer disease outcomes. For example, a study of DENV-patients showed that those with higher frequencies of circulating pDCs displayed less severe disease and had lower viral loads [141]. Viruses have evolved a multitude of ways to evade antiviral sensing by infected cells, but the cell–cell transfer of virus from infected cells to pDCs makes them especially suited to counteract these viral evasion mechanisms [142]. There is still a lack of evidence regarding the role of pDCs in the skin during tick-borne viral infections. Investigating the dynamics of pDC seeding in the skin during tick feeding and CCHFV transmission, as well as the effect of tick saliva on pDCs, is of future research interest.

#### 5.3.3. Monocytes

Monocytes are accessory cells that link innate and adaptive immunity and are involved in the response to infection. They have an array of PRRs and scavenger receptors that sense microbes and apoptotic cells and constantly survey the body, reacting rapidly to pathogen invasion by releasing potent effector molecules depending on the monocyte subset involved. Three monocyte subsets have been defined so far. The first subset, classical monocytes, is delineated by the high expression of CD14^+^ and the absence of CD16. Classical monocytes express pro-inflammatory cytokines and chemokines, including IL-6, IL-8, TNF-α, and IFN-γ, and are involved in the phagocytosis of viruses and virus-infected cells. This subset can also mediate direct killing by producing reactive oxygen species. Under inflammatory conditions, such as those induced by infection, classical monocytes infiltrate the skin where they differentiate into Mφ and monocyte-derived DCs (moDCs) [143,144]. The second subset, non-classical monocytes, are CD14^−^CD16^+^ and display anti-inflammatory properties. This subset has been observed in close association with the vasculature, where they clear dying endothelial cells [145,146,147]. The last subset, Intermediate monocytes, are CD14^+^CD16^+^ and play a role in antigen presentation [148,149].

Under inflammatory conditions, monocytes rapidly accumulate in the skin from the circulation in response to cytokine and chemokine gradients produced by other cutaneous cells. Monocytes exert direct antiviral activity through phagocytosis and the production of nitric oxide (NO), and indirectly by producing proinflammatory cytokines that amplify the local immune response [150,151]. These cells differentiate into moDCS or Mφ, which serve to further augment the local cutaneous response to inflammatory stimuli. Upon the resolution of infection, monocytes contribute to tissue repair by producing growth factors and clearing apoptotic cells and debris, highlighting their important role in mediating immune homeostasis. The acquisition of this regulatory phenotype is partly dependent on the tissue microenvironment [152].

Monocytes and moDCs are permissive to CCHFV and are productively infected; they are considered the primary target of the virus [7,153,154,155]. Monocytes are thought to contribute to the dissemination of CCHFV to distal organs due to their ability to traffic through blood vessels, essentially functioning as a Trojan horse. Severe cases are characterized by high viral loads, monocytosis, lymphopenia, and the presence of CCHFV antigens in tissues at autopsy [156,157,158]. Investigating the mechanisms underlying the monocyte response to CCHFV and how the virus shapes monocyte activation and fate could provide insight into why some infections progress to severe, life-threatening CCHF.

#### 5.3.4. Macrophages

Mφ are a functionally diverse self-renewing subset of MPCs involved in pathogen detection and removal, chemoattraction of other immune cells, wound repair, and tissue homeostasis in the skin. Mφ reside deeper in the dermis, in perivascular regions and have potent phagocytic and microbicidal activity, directly mediating pathogen removal by engulfing and destroying viruses and virus-infected cells [159,160]. Mφ polarization is driven by microenvironmental stimuli, such as damaged or infected cells in surrounding tissues and cytokines released by activated immune cells. Mills et al. [161] proposed the classification of macrophages into two subsets, M1, or classically activated, and M2, or alternatively activated Mφ. This classification was based on the observation that arginine was metabolized differently depending on the dominant cytokines produced by T cells in different mouse strains. M1 Mφ exhibit proinflammatory activity and are polarized by granulocyte-monocyte colony-stimulating factor (GM-CSF), lipopolysaccharide (LPS), and IFN-γ, while M2 Mφ are considered anti-inflammatory and are polarized by IL-4 and TGF-β. Recent studies, however, have shown that Mφ exhibit remarkable plasticity, and multiple subpopulations are now recognized. M2 Mφ are further classified into M2a, M2b, M2c, and M2d based on their gene expression and cytokine secretion profiles [162,163].

Activation of Signal Transducer and Activator of Transcription 1 (STAT1) and Interferon regulatory factors (IRF) pathways by IFNs and TLR binding polarizes Mφ to the M1 phenotype. M1 Mφ are involved in the response to viral pathogens and produce copious amounts of proinflammatory cytokines, including TNF-α, IL-1β, IL-6, and IL-23, and proinflammatory factors such as nitric oxide synthase (iNOS) and ROS. Upon activation, M1 macrophages upregulate the expression of surface markers such as CD80 and CD86, costimulatory molecules, and MHC II, which are involved in T cell activation.

STAT 6 activation by IL-4 and IL-13 polarizes Mφ towards an M2 phenotype [164]. Arginase production is a defining characteristic of M2 macrophages, and hypoxia inducible factor (HIF-2α) and the cytokine IL-21 have been shown to contribute to M2 differentiation by reducing NOS2 expression [165,166]. M2 Mφ are involved in tissue repair and wound healing, and in addition to arginase 1, express IL-10, transforming growth factor (TGF)-β, vascular endothelial growth factor (VEGF), and epidermal growth factor. Increased expression of the surface markers CD163, C204, and CD206 is also used to define M2 Mφ. CD163 and CD204 are scavenger receptors involved in the clearance of damaged cells from the sites of infection and induction of anti-inflammatory cytokines, while CD204, an acetylated low-density lipoprotein receptor, binds apoptotic cells [167,168]. CD206 is a mannitol receptor involved in viral antigen uptake and processing [169].

The Mφ response to infection requires a fine and intricate balance. During the early stages of pathogen invasion, Mφ are skewed to the M1 phenotype. M1 macrophages mediate a pro-inflammatory response that enables pathogen elimination and the removal of damaged cells. Toward the end of the inflammatory phase, M2 macrophages are necessary to resolve inflammation, repair and regenerate tissue, and return the body to homeostasis.

A feature of viral hemorrhagic fevers, such as CCHF, is the aberrant overproduction of proinflammatory cytokines, leading to a dysregulated immune response. This phenomenon is often referred to as a cytokine storm and is akin to what has been observed during septic shock. A study in a septic mouse model showed that immunoregulatory CD204^+^ Mφ were necessary to suppress the proinflammatory response and ameliorate septic shock [170]. This study highlighted the importance of M2 macrophages in restraining an overactive inflammatory response.

Dermal Mφ treated with IL-4 have been shown to be permissive to DENV, enhancing viral replication [171]. Mouse and human M1 Mφ appear to be refractory to CCHFV while M2 macrophages can be infected; however, it is not yet known whether M2 Mφ are productively infected, and the impact of M2 infection on viral pathogenesis remains to be investigated [154]. Further investigations into the roles of Mφ subsets in tick-borne viral infections could shed light on their pathogenesis, thereby laying the groundwork for studies that target appropriate signaling pathways to modulate macrophage function.

#### 5.3.5. Lymphocytes

##### T Cells—Deciding Viral Fate?

Tissue-resident lymphocytes in the skin include T cells, B cells, NK cells, innate lymphoid cells (ILCs), and mucosal-associated invariant (MAIT) T cells. Generally, T cells express either αβ or γδ T cell receptors (TCR). These receptors are coupled to the CD3 protein complex, forming the complete antigen-binding TCR. Studies in mice have shown that mφ, ILCs, γδ T cells, and NK cells are endowed with tissue-resident properties during embryogenesis [172].

αβ and γδ T cells are present in both the epidermis and dermis, but in normal human skin most of these cells are found at the interface between the epidermis and dermis and near blood vessels. αβ T cells comprise most T cells present in the skin and acquire their tissue-resident properties in their effector stages following pathogen encounter. The activation of αβ T cells requires recognition of antigens presented in the context of MHC molecules and a secondary signal resulting from the interaction of co-stimulatory molecules on T cells with their corresponding ligands on APCs [173]. The release of cytokines by APCs and other cells in the immediate microenvironment constitutes the third signal and is key for the differentiation of T cells into diverse effector subsets thus determining their specific function [174,175].

γδ T cells constitute between 1–10% of total T cells in the skin and display characteristics of both innate and adaptive immune cells [176,177]. γδ T cells express TLRs and an NK cell receptor (NKG2D) and can mediate direct cytolysis, and, unlike αβ T cells, antigen recognition can occur independently of MHC molecules [178]. They function as APCs, processing and displaying antigenic fragments on their surface. This includes both direct presentation of antigens and cross-presentation, which enables them to activate αβ T cells [179]. In contrast to αβ T cells, γδ T cells only require a singular signal to become activated, i.e., antigen recognition by the TCR. They can recognize a wide variety of antigens encompassing both self- and non-self-antigens and, in addition to peptidic molecules, can also recognize lipids, heat-shock proteins, and phosphoantigens [175,180]. Upon activation, γδ T cells release cytotoxic molecules that directly kill virus-infected cells and produce cytokines such as IFN-γ, IL-17, and IL-22 that activate the surrounding immune cells [175].

T-cells are fundamental components of the adaptive immune response and mediate protection against various viruses. There are 3 distinct subtypes of T-cells that shape the immune response and are delineated based on the expression of cell surface molecules. The first and second subtypes are defined by the expression of CD4 and CD8. The third subtype, T_reg_, is defined by the expression of CD4, CD25, and FOXP3 [181].

CD4^+^ T cells, also termed helper T cells, bolster the immune response by activating CD8^+^ T cells and other immune cells and providing important signals to B cells that induce their differentiation into antibody-producing plasma cells [182,183]. Activated CD4^+^ T cells differentiate into distinct T helper subsets based on the cytokine milieu in the surrounding environment and are defined by secretion of signature cytokines, effector roles, and lineage-specific transcription factors [184,185]. They include T_H1_, T_H2_, T_H17_, T_H9_ and T_H22_, and T follicular helper (T_FH_). Of these, T_H1_, T_H2_ and T_H17_ are involved in the response to pathogens. In the context of viral infections, T_H1_ cells drive type 1 immunity, support CD8^+^ T cell cytotoxicity, and contribute to protection by secreting IFN-γ and other proinflammatory cytokines that activate other immune cells. T_H2_ cells orchestrate immunity against large parasites and are characterized by their secretion of IL-4, IL-5, and IL-13. Skewing towards type 2 immunity is associated with impaired antiviral defense. IL-4 and IL-13 produced by T_H2_ cells in the skin suppress the production of key AMPs, including LL-37, and disrupt the barrier function of keratinocytes by opening tight junctions [186,187,188].

CD8^+^ T cells, also termed killer T cells, are essential for viral clearance and are involved in immune-mediated cell death [130]. During primary infections and following priming, activated CD8^+^ T cells become cytotoxic, producing perforins and granzymes—serine proteases that induce caspase-dependent and—independent apoptotic pathways [189]. They migrate to the infection site where they exert their cytotoxic function, thereby curbing viral spread. Activated CD8^+^ T cells produce IFN-γ, creating an antiviral state that is inconducive to pathogen survival and which contributes to shaping myeloid and CD4^+^ T cell function [190,191]. In the context of CCHFV, CD8^+^ T cells have been found to play a role in viral clearance and were crucial to disease control in a susceptible mouse model of CCHFV [192].

T_reg_ play a vital role in immune homeostasis by maintaining tolerance and suppressing unrestricted immune responses mediated by effector T cells [193]. They produce inhibitory cytokines such as IL-10 and TGF-β, which dampen the inflammatory response, and outcompete effector T cells for IL-2, depriving them of a key nutrient, resulting in lower proliferation [194,195,196,197]. Tregs mitigate the extent of immunopathological damage during localized infections by viruses such as ZIKV and HSV in the skin and RSV in the lungs and have been shown to be necessary for the priming and migration of CD4^+^ T cells after infection with HSV [198,199,200]. Severe CCHF is characterized by dysregulated immune response, coagulation abnormalities, vascular pathology, and multi-organ failure. Severe cases display elevated proinflammatory cytokine levels and immune cell hyperactivation [37,201,202]. T_reg_ may be important modulators of the aberrant response associated with severe CCHF, and investigating their role may help identify specific signatures that correlate with outcome. Defining the kinetics, phenotype, and functional capacity of Tregs across the CCHF disease course will be essential to understanding why some patients progress to fatal hyperinflammation.

##### Memory T Cells

Long-term peripheral immunity to viral infections is mediated by migratory and T_RM_ [203,204,205]. Following priming in the draining lymph nodes, T cells become activated and acquire an effector state characterized by the modulation of different markers associated with tissue ingress.

T_RM_ are characterized by the expression of CD69, CD103, and CD49a. CD103, an αβ integrin, is expressed by CD8 T_RM_ and can be considered the definitive marker of these cells. In contrast, CD69, a CLR, is also expressed by circulating T cells and can be used to delineate activated T cells. CD4 T_RM_ are identified by the expression of both CD69 and CD103, as not all subsets express CD103 [206]. TRMs in the skin are functionally and clonally heterogeneous and have distinct trafficking and cytokine secretion properties [207,208].

Activated T cells undergo migrational imprinting in the draining lymph nodes (dLNs), which facilitates homing to the sites of infection. Following activation, T cells upregulate E- and P-selectin ligands, including cutaneous lymphocyte-associated antigen (CLA), together with chemokine receptors, including CCR4, CCR8 and CCR10, which facilitate tethering, rolling, and arrest on dermal vessels, thus enabling their extravasation into infected tissue [209].

Early after priming, activated CD8 T cells acquire different phenotypes that determine their fate following the resolution of infection. Short-lived effector cells (SLECS) display high cytotoxicity and are characterized by high expression of KLRG1, while memory precursor effector cells (MPECs) express low levels of KLRG1 and higher levels of CD127. About 90% of effector CD8^+^ T cells undergo apoptosis after mediating pathogen clearance, leaving MPECs, which continue to form a pool of long-lived memory cells that mediate protection to secondary infections [210,211].

Studies have demonstrated that CD8^+^ T cells expressing KLRG1 can persist for months following acute infection and that these cells, termed long-lived effector cells (LLECs), maintain distinct migration properties, function, and proliferative potential [212]. In a study by Lucas et al. [212], LLECs were found to be capable of migrating from circulation to non-lymphoid tissue, where they acquired a transcriptional program like T_RM_. This process was dependent on antigen presentation by endothelial cells, and migrated LLECs significantly reduced the pathogen burden, highlighting their importance in mediating protection against secondary infections.

T_RM_ were once thought to be stationary, but recent studies have revealed that they actively patrol the skin and can interact with LCs in the epidermis [213]. Using intravital multiphoton microscopy, Gebhardt et al. [204] showed that following HSV infection in mice, CD4^+^ T cells localized to the dermis while CD8^+^ T cells localized to the epidermis. Following the resolution of infection, HSV-specific memory T cells consisted of slow-moving CD8^+^ T_RM_ at the site of infection and CD4^+^ T_RM_ that recirculated rapidly through the dermis [204]. Another study demonstrated that following acute VACV skin infection, the generated CD8^+^ T_RM_ were retained in the skin where they persisted for up to 6 months. These cells populated not only the site of infection but were found in other skin surfaces [214].

T_RM_ can proliferate rapidly in response to infection and are uniquely positioned to quickly attack virus-infected cells. Immune responses mounted against tick proteins, and the generation of tick protein-specific T_RM_ may be beneficial to the host during subsequent encounters with ticks and during pathogen transmission. CCHF is prevalent in regions where livestock farming is essential to the economy. Infection in livestock is subclinical, and farmers, herders and abattoir workers in endemic areas are at particularly high risk as they are continually in contact with animals. This is further compounded by heavy exposure to ticks. In the event of secondary infections with CCHFV, we envision that T_RM_ could rapidly act and boost the local response by driving a robust IFN-mediated proinflammatory response at the tick feeding site, which may reduce the chances of viral dissemination and systemic infection.

##### Cutaneous B Cells—Uncovering New Frontiers

Although present, B cells are sparse in healthy human skin, and little is known about their function in the skin [215,216]. In certain inflammatory conditions, there is an increase in the cutaneous B cell population. Cutaneous B cells are poorly characterized, and the mechanisms underlying B cell recruitment to the skin are largely unknown. It is also not clear whether the B cells in the skin are truly tissue-resident or if they are migratory and infiltrate the skin during inflammatory processes.

B cells are a key component of the adaptive immune response and play key effector roles in the immune response to pathogens. These cells can directly block viral entry by secreting antibodies that block virus binding sites, thereby interfering with virion attachment to cellular receptors [217,218]. Antibodies can also form immune complexes with virus particles, rendering the particles ineffective by interfering with their ability to engage receptors. Antibodies indirectly block viral spread through FC effector mechanisms and opsonization, leading to antibody-dependent cell cytolysis by NK cells, antibody-dependent cellular phagocytosis by Mφ, and complement-mediated cytolysis by complement molecules [219]. In addition to antibody secretion, B cells play a role in T cell co-stimulation, antigen presentation, and the production of both pro-inflammatory and anti-inflammatory mediators [220].

In the context of CCHFV, survivors have been shown to mount a robust humoral response, and interestingly, both neutralizing and non-neutralizing antibodies are protective and correlate with survival and vaccine efficacy [221,222]. Non-neutralizing antibodies to the CCHFV nucleoprotein dominate the response and are correlated with survival, contrary to other hemorrhagic fever viruses where neutralizing antibodies to the glycoprotein are essential for protection [221,223]. Longitudinal studies have shown that survivors mount a durable response to the nucleoprotein, GP38, and Gc up to a decade after acute infection [218,224,225].

There is mounting evidence that B cells can produce antibodies locally in the skin. IgM, IgG1, IgG2, and IgG3 were detected in healthy human skin, pointing to a potential role in the early defense against pathogens [226,227].

Evidence for human cutaneous B cell responses remains scarce, and no studies have directly examined their contribution to local immunity during or after CCHFV infection. Elucidating how skin-associated B cells participate in the response to CCHFV may help explain why some individuals successfully control infection whereas others develop fulminant disease.

#### 5.3.6. Innate Lymphoid Cells in the Skin

Other cell types that contribute to antiviral immunity include innate lymphocytes (ILCs), which comprise five subsets including NK cells, ILC1s, ILC2s, ILC3s and lymphoid tissue inducer (LTi). NK cells and ILC1s are cytotoxic and can directly kill virus-infected cells. NK cells circulate in the peripheral blood but can migrate to tissues, such as the skin, in response to inflammatory cues. They are involved in the direct elimination of pathogens via cytotoxic granules and indirectly by activating FAS and TNF—death receptors that trigger a signaling cascade leading to the apoptosis of infected cells. They produce IFN-γ and TNF-α, which activate other skin-resident immune cells [228]. Like Mφ, ILCs are plastic and can modify their phenotype based on the local cytokine milieu, allowing them to adapt and coordinate antiviral defense in the skin [229,230].

Skin biopsies from acute DENV patients were found to have activated CD56^bright^ NK cells that expressed homing markers specific to the skin. Higher levels of IL-18 were detected in skin blisters and the plasma of these patients, pointing to the involvement of an IL-18-dependent mechanism in the DENV NK cell response [231]. IL-18 promotes Th1 immunity and induces CD8^+^ T cells, NK cells, and DCs to produce IFN-γ [232,233]. CD8^+^ T cells are especially important and have been implicated in viral clearance. In the presence of IL-12, IL-18 can induce IFN-γ secretion by antigen-experienced memory CD8+ T cells in the absence of the antigen [234].

NK cells were found to be significantly higher in patients with severe CCHF and fatal cases compared to those with a milder course of the disease [235]. The temporal and spatial dynamics of these cells remain to be elucidated, and further studies are required to determine the role of NK cells in skin antiviral immunity against CCHFV and other tick-borne viruses. NK cells and ILCs are the subject of an excellent review by Johnson et al. (2025) [228] and Piersma et al. (2024) [236].

#### 5.3.7. Fibroblasts—Beyond Structure

Fibroblasts are stromal cells that synthesize collagen and ECM [237]. They are of mesenchymal origin and are found in various organs, including the skin, where they help maintain structural integrity [61]. Fibroblasts display functional and morphological heterogeneity and are involved in wound healing, tissue remodeling, and cell signaling [237,238]. They are present at distinct anatomical sites where they play a vital role in tissue synthesis and regeneration after damage or injury. Initially, they were thought to only be involved in the structural maintenance of organs, but their role in regulating immunity has become apparent and is increasingly appreciated. They have recently been shown to directly participate in the innate immune defense to pathogens by synthesizing antimicrobial peptides, secreting cytokines, and recruiting leukocytes [239,240]. Two major groups of fibroblasts were initially described in the human dermis based on gene and protein expression. A meta-analysis of human dermal transcriptomic data led to the identification of three major fibroblast groups using single-cell RNA sequencing [241,242]. These groups are demarcated based on the expression of 3 genes, designated as A, B, and C. Group A appears to be involved in tissue homeostasis, while group B appears to be involved in immune surveillance. Type C fibroblasts are found in the deep dermis and hypodermis and express genes associated with ECM organization and modelling [62].

Fibroblasts express functional surface, endosomal, and cytosolic TLRs, NLRs, and RLRs that they use to sense viral molecules [243]. The binding of specific skin PRRs by their cognate ligands leads to the initiation of innate immune responses, including the secretion of cytokines such as IL-6 and TNF-α and chemokines such as CXCL8, a neutrophil chemoattractant, and CXCL9, CXCL10, and CXCL12, which promote the migration of leukocytes [239]. Fibroblasts are activated by Type 1 IFN, TNF-α, IL17A, IL-1α, and IL-1β and respond by upregulating the expression of key leukocyte-attracting chemokines and secreting proinflammatory cytokines that are crucial to viral defense [239]. In addition, they produce β-defensins, which, together with the proinflammatory cytokines they secrete, induce an antiviral state, thereby protecting neighboring cells [239,244]. Interestingly, the stimulation of skin fibroblasts with IL-17A induces the production of G-CSF and GM-CSF, which induce keratinocyte proliferation and epidermal differentiation [245,246,247]. GM-CSF also influences macrophage polarization [247].

Studies of virus-tick feeding in mouse models have shown that fibroblasts are the early targets of Powassan virus (POWV) and tick-borne encephalitis virus (TBEV) [248,249]. They have also been demonstrated to be permissive to a range of arboviruses, including Chikungunya virus (CHIKV), ZIKV, WNV and DENV [84,240,250,251]. ZIKV infection of human dermal fibroblasts was recognized by RIG-I and MDA-5, resulting in the initiation of IFN signaling and cytokine-cytokine interaction pathways, and the knockdown of RIG-I resulted in the suppression of antiviral responses and higher viral replication in these cells. The addition of RLR agonists resulted in a lower infection rate [240]. In an in vitro fibroblast coinfection model of ZIKV and DENV, transcriptomic analyses revealed that prior DENV infection inhibits ZIKV infection by inducing an antiviral state through the upregulation of innate immune pathways, the suppression of the ZIKV receptor, and the downregulation of genes involved in clathrin-mediated endocytosis [252].

Primary skin fibroblasts are permissive to CCHFV strain IbAr10200, but infection results in low titers [253]. The effect of infection on these cells has, however, not been extensively explored. In some vector-borne viral infections, such as Toscana virus (TOSV), which is transmitted by sandflies, fibroblasts have been found to drive viral amplification. Sandfly saliva was shown to reprogram fibroblasts into a wound healing state, increasing their permissiveness and enhancing viral replication [254].

The important role that skin fibroblasts play in recognizing and responding to viruses is increasingly becoming clear. Investigating the interplay between fibroblasts and immune cells, and their contribution to the overarching cutaneous immune response, may provide crucial insights into disease pathogenesis and may reveal strategies to curb viral dissemination during CCHFV infection.

#### 5.3.8. Granulocytes

Granulocyte involvement in tick-borne viral infections of the skin is mainly indirect, through signaling and inflammation. In the steady state, granulocytes circulate in the blood but infiltrate peripheral tissues during inflammation. Following tick attachment, mast cells, basophils, eosinophils, and neutrophils congregate at the bite site in response to tick saliva and the resultant tissue damage that occurs during feeding. This creates an inflamed, saliva-modulated environment that may favor or hinder viral replication. Basophils and mast cells mediate anti-tick immunity by remodeling the cutaneous microenvironment. Repeated tick bites have been shown to lead to acquired tick resistance (ATR), which is partly mediated by basophils and mast cells [255,256]. Basophils and mast cells possess high-affinity IgE receptors (FceR1), which bind to IgE. Cross-linking of IgE on the surface of these cells triggers degranulation and histamine release [257,258]. Histamine induces inflammation and vasodilation of blood vessels, drawing immune cells to the bite site. In mice, intravital fluorescence imaging of tick bite sites revealed that basophils cluster in the epidermis around the tick mouth parts, and depletion of these cells resulted in the abrogation of ATR [259]. Basophils and mast cells shape the cutaneous microenvironment and may potentially influence local viral replication and spread. There are no studies on the roles of basophils and mast cells during CCHFV infection, and the contribution of these cells to ATR in humans has not been delineated.

Neutrophils are among the first cells recruited to tick bite sites and are strongly influenced by arthropod saliva. They are critical in shaping early inflammation and exert effector functions that limit viral spread. Such effector functions include phagocytosis, cytokine production, ROS production, and Neutrophil extracellular Traps (NETs), termed NETosis [260]. Neutrophil activation can have deleterious effects on the host, as the effector molecules they secrete can promote endothelial dysfunction and, indirectly, enhance viral dissemination through signaling/inflammation [261,262]. Neutrophil-driven inflammation in the skin facilitates the retention of the virus and the recruitment of permissive immune cells during Semliki Forest virus (SFV) infection, where recruited myeloid cells become infected and amplify the virus [263]. Investigating the contribution, if any, of neutrophils to the cutaneous response to CCHFV may shed light on its pathogenesis.

## 6. Crosstalk in the Skin—Strength in Diversity?

Immune skin cells interact under inflammatory conditions to form lymphoid tissues known as the skin-associated lymphoid tissue (SALT), a conceptual framework proposed by Streilein in 1983. This was based on observations that cells in the skin can recognize, process, and present antigens; skin draining lymph nodes can respond to immunogenic signals; T cell subsets can differentiate and home to the skin, and that T cell homing is reliant on differentiation signals produced in situ by other cutaneous cells. Natsuaki et al. [264] described the formation of transient leukocyte-clustering structures comprising DCs, macrophages, and T cells in the skin and termed them inducible skin-associated lymphoid tissue (iSALT). More studies have demonstrated the importance of iSALT in response to inflammatory stimuli and function as sites for antigen presentation [73,265,266].

Understanding the interactions between various immune cell subsets and non-immune cells is paramount to determining the mechanisms underlying immune reactions to microbial insults. These interactions can have beneficial or deleterious effects on the host. Cutaneous cells work together to form an intricate system that can limit viral replication and dissemination. Keratinocytes have tight junctions that form a physical barrier and prevent viral entry. When infected, they produce antimicrobial peptides and proinflammatory cytokines that initiate the innate immune response. Indeed, in mice, CCL-27 produced by keratinocytes induces the upregulation of CCR-10 on circulating CLA^+^ T cells, facilitating their recruitment to inflamed skin regions [267]. In humans, CCL-1 produced by keratinocytes in the epidermis functions as a chemoattractant to T cells and induces the expression of CCR-8 and CLA, which instruct T-cell homing [268].

LCs and DCs act as sentinels, constantly surveilling the skin for foreign invaders. Upon antigen encounter, they migrate to the skin-draining lymph nodes where they present processed antigens to T-cells, thereby activating the adaptive immune system. When activated, lymph node-resident T-cells upregulate migratory markers, enabling homing to the skin where they mediate a robust immune response to control pathogen replication. In the event of secondary exposure, T_RM_ rapidly expand and halt viral replication by directly killing pathogen-infected cells or by producing proinflammatory cytokines that contribute to the establishment of an antiviral state and the recruitment and activation of more immune cells. LCs interact closely with DCs and can transfer antigen-MHCII complexes following infection by HSV [269].

Epaulard et al. [270] showed that in cynomolgus macaques, targeting HIV gag or Influenza hemagglutinin (HA) to LCs led to virus-specific immune responses, and that Mφ and neutrophil-derived TNF-α further enhanced the response when Poly I:C was used as an adjuvant.

An elegant example of immune cell crosstalk involves interactions among APCs, basophils and mast cells, CD4^+^ T cells, B cells, and keratinocytes. During feeding, ticks inject various molecules during feeding which are taken up by APCs. Activated APCs migrate to the dLNs, where they prime naive T cells, which in turn become activated and differentiate into Th subsets. Of these, T_H2_ cells produce IL-4, which activates B cells and promotes their differentiation into IgE-producing plasma cells. Tick antigen-specific IgE arms basophils and mast cells by binding to their FceR1. During secondary feeding, these basophils emigrate from the blood into the skin, where they become activated, degranulate, and release histamine. In addition to increasing vascular permeability and triggering inflammation at the bite site, histamine induces the proliferation of keratinocytes, leading to epidermal hyperplasia, which was shown to inhibit blood feeding in mice [259,271,272].

IL-3 produced by cutaneous CD4^+^ T_RM_ has been shown to enhance basophil adhesion to the endothelium and promote basophil extravasation through the endothelium to tick bite sites [273,274]. Basophil recruitment to the skin was impaired in mice that lacked T cells but was restored when CD4^+^ T_RM_ from tick-resistant mice were adoptively transferred, highlighting the importance of crosstalk between these two cell types [275].

The network formed by cutaneous cells is complex, and the interplay between these cells is layered, multifactorial, and adaptable, involving fine-tuned communication between diverse cell types that collaborate to protect the host against viral infections. The skin also contains diverse and non-pathogenic microorganisms that interact with epithelial and immune skin cells and instruct the immune system. These interactions are tightly regulated and contribute to the maintenance of immune homeostasis [276,277].

Viruses have evolved ways to evade the immune system and even hijack immune cells to ensure their survival. The first round of infection occurs in the epidermis upon pathogen entry, after which the initial target cells produce inflammatory mediators that recruit susceptible migratory distal immune cells that can contribute to systemic viral spread [278]. Indeed, infected keratinocytes, the major cell type infected by DENV, contribute to viral propagation by drawing myeloid cells to the site of infection [279]. DENV can block type 1 IFNs, thereby allowing unrestricted replication and enhancing viral spread facilitated by infected myeloid cells migrating out of the skin [280]. CCHFV encodes proteins that also function as IFN antagonists and is suspected of using monocytes and DCs to facilitate its dissemination to distal organs such as the spleen, liver, and kidneys.

The site of infection could be an important determinant of subsequent systemic disease course and clinical outcome. Modulating the local skin immune response to suppress viral replication can reduce the probability of the systemic spread of CCHF. Developing vaccines or interventions that train local skin immune cells, enhance their interaction, rebalance the antiviral immune response, or engage PRRs on cutaneous immune cells represent attractive strategies. Conversely, ensuring a well-regulated inflammatory response in the skin could avoid the overactivation of immune cells and the overproduction of mediators that mediate tissue damage. Functional modulation of cutaneous immune cells and their impact on tick-borne pathogens warrants further investigation.

## 7. Dissecting Cutaneous Immunity—In Search of the Perfect Model

**Figure 2:**
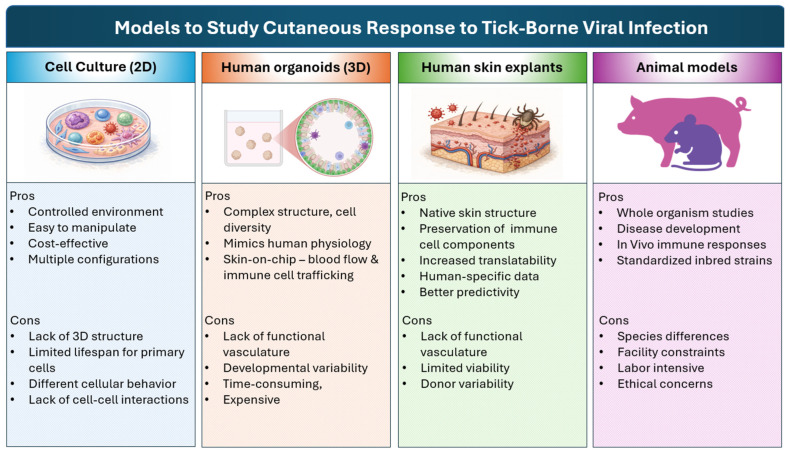
Models to study the cutaneous response to tick-borne viral infections. Comparison of the advantages and disadvantages of systems used to investigate vector-borne viral infections.

The cutaneous immune response can be studied from a variety of perspectives, using several model systems (Figure 2). From the most basic system, such as 2D cell culture, to the more in-depth in vivo models, such as rodents, pigs, and non-human primates (NHPs). However, there are a variety of options in between, including human organoids, which offer a more complex system for mimicking human physiology, and ex vivo tissue cultures such as human skin explants, which preserve the native cellular architecture and structures of human skin (Figure 2). Despite the availability of these intricate model systems, in vivo models are still widely used in pathogen research.

In vivo models enable the study of complex biological systems and facilitate investigations into interactions between cells, tissues, and organs, providing crucial insights into the mechanisms underlying disease pathogenesis. Animal models have contributed immensely to the advancement of biomedical research and are foundational to vaccine and drug development. Although they are still widely used and have demonstrated significant value, a key challenge remains: their limited translatability. Animal models, especially laboratory animals such as inbred mice and rats, do not entirely replicate the complexity and variability of humans. This has resulted in poor predictability for vaccine and therapeutic safety and response. Additionally, physiological, metabolic, and genetic differences limit the extent to which data can be extrapolated and interpreted, especially for organs that differ significantly across species, such as skin. Indeed, human, rodent, NHP and even pig skin differ not only structurally but also immunologically. For example, as in humans, mouse skin contains Mφ, LCs, DCs, and T cells, in addition to dendritic epithelial T cells (DETCs), which are absent in humans. This specialized cell subpopulation participates in immune surveillance and skin homeostasis [276,281]. There are also differences in the distribution of skin-resident immune cells that impact the immune response to injury or infection. Rodents lack a multi-layered epidermis as well as the papillary, reticular, and hypodermal regions of the dermis [276,282]. Additionally, human skin contains immune microenvironments that rodents lack [283] and is also less permeable, which adds another obstacle to the translatability of drug studies that rely on topical application.

Humanized mouse models have been proposed to address this issue. They have been used for disease modelling and mechanistic studies of vaccine responses and drug interactions. Irradiated immunodeficient mice engrafted with human hematopoietic stem cells (HSCs) can reconstitute their hematopoietic system, resulting in mice with functional human immune systems [284]. Various mouse strains with varying degrees of immune deficiency have been used to develop these models. The use of immunodeficient mice allows for a better engraftment efficiency but also results in impairments and/or defects in cytokine responses and B and T-cell, DC, Mφ, and monocyte function depending on the mouse strain used [285,286,287,288,289].

Agarwal et al. [282] developed a mouse and rat model containing cutaneous immune cells for the study of skin infections by engrafting full-thickness human skin, autologous lymphoid tissue, and autologous stem cells into immunodeficient mice. These humanized rodent models developed both innate and adaptive immune cells and supported the development of spleen and thymus, lymphoid organs necessary for the initiation of pathogen-specific immune responses [282]. Klicznik et al. [290] generated a humanized mouse model for the study of human T cell recruitment and dynamics using engineered skin from human keratinocytes, fibroblasts, and autologous peripheral blood mononuclear cells (PBMC). These studies demonstrated that humanized immune system mice can facilitate the study of immune responses to skin-tropic infections. A drawback of such models is that generating them is laborious and expensive.

The supplementation or replacement of such animal models with bioengineered tissue or explants can enhance translatability and lessen the impact of species-specific genetic differences. Most investigations into cutaneous immune responses to infection concentrate on individual cell types. A comprehensive understanding of the mechanisms underlying immune responses to pathogens requires examining the dynamic interplay between immune and non-immune cells, as well as the contribution of the stromal compartment in modulating immunity.

Human skin explants have emerged as an alternative to rodents for skin disease modeling. One advantage is that they preserve the complex native architecture of the skin, including immune cells and the extracellular matrix. They can also be used to assess individual variability across different patients and, if used on a large scale, could provide insight into the underlying genetic differences involved in predisposition or susceptibility to disease. In the context of vector-borne, skin-borne, and skin-tropic viral disease, the use of skin explants has contributed to the identification of skin cells permissive to Ebola virus (EBOV), Usutu virus (USUV), WNV, Mayaro virus (MAYV), and ZIKV [291,292,293].

## 8. Ex Vivo Approaches to Studying Tick-Host Interactions

For the past 50 years, artificial feeding systems have been developed not only for tick rearing and colony maintenance, but also for pathogen research. However, more recently, artificial feeding systems have been extended to capture the effect of arthropod feeding and pathogen injection in relevant models, such as human or animal skin, using sophisticated systems.

Ticks require days to weeks to complete a feeding cycle, complicating the use of human skin explants. Some complications include lack of vasculature, willingness to feed, and viability of the explant throughout feeding. While human skin has not been used as a membrane for artificial feeding or probing for ticks, there are several publications that utilize animal skin, which is intended to mimic live animals, allowing for a more innate feeding response [294,295,296]. To address the complications, the connection of a peristaltic pump could allow for filling of the vasculature and mimic natural host rhythms. Ticks can be coaxed to feed by introducing a variety of stimuli, including volatile compounds. Additionally, ticks that have already begun feeding on an animal or other artificial feeding systems are more willing to re-attach for feeding (unpublished, personal communication). For the viability of human skin explants, several studies have reported viability for weeks [297,298] to months [299], allowing ample time for attachment and attempted feeding. With this model system, the transmission of CCHFV from infected ticks and the dynamics of the cutaneous responses could be elucidated.

## 9. Knowledge Gaps in the Cutaneous Response to CCHFV

There are several key gaps that exist in our understanding of how CCHFV interacts with both the ticks and the host and what ensures a conducive environment for productive infection. This has limited the rational design of vaccines and therapeutics that leverage cutaneous immunity to restrict systemic viral spread.

### 9.1. What Unfolds in the Skin During a Tick Bite?

**Figure 3:**
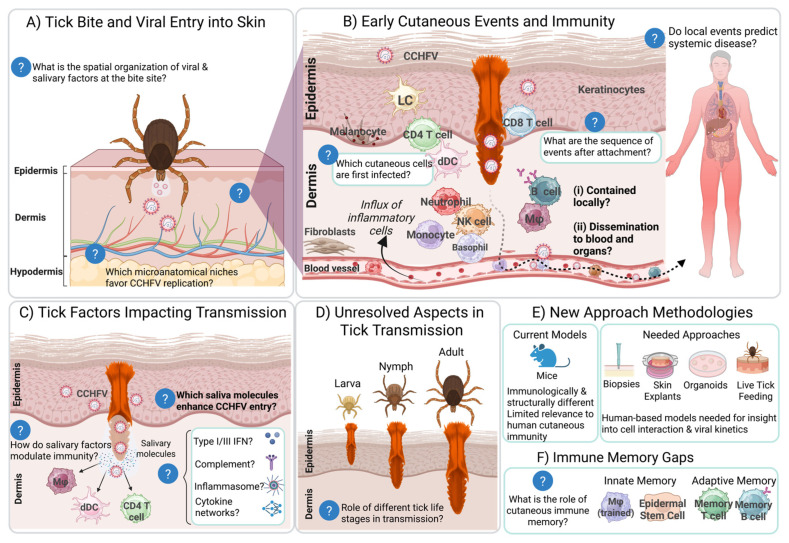
Illustration of the key knowledge gaps in the cutaneous response to CCHFV at the tick–host–virus interface. (**A**) Tick bite and viral entry into skin; (**B**) Early cutaneous events and immunity; (**C**) Tick factors impacting transmission; (**D**) Unresolved aspects in tick transmission; (**E**) New approach methodologies; (**F**) Immune memory gaps. Created in BioRender. Burch, M. and Olal, C. (2026) https://BioRender.com/nozff6g.

The skin serves a dual role, acting as a barrier to viral transmission while also serving as a reservoir for viral replication and dissemination. There is a dearth of studies that directly characterize the skin virome after tick bites, and no high-dimensional omics studies of early bite-site occurrences. Additionally, the spatial organization of salivary factors and viral particles at the bite site is unknown (Figure 3). The skin has microanatomical niches in which immune and non-immune cells interact and coordinate various biological processes, including host defense [300,301]. The tick-mediated damage that occurs during tick feeding and the resulting inflammation determine which cells are involved and will interact during virus transmission. These compartments likely provide a conducive environment for viral replication and persistence and these niches, if any, have not yet been identified for CCHFV. Do chronic cutaneous sequelae exist after CCHFV infection? What occurs in these inflammatory compartments? Could these serve as a focal point from which the disease recrudesces? To what extent does the local immune response play a role in the cutaneous manifestation observed during CCHFV? The use of integrated multi-omics may provide a deeper understanding of the cell types involved in coordinating proper immunity while revealing those that exhibit dysfunctional behavior.

The sequence of events that occur following tick attachment and the transmission timeline have not yet been defined for CCHFV. The functions of the initial target cells of CCHFV in intact human tissue have also not yet been fully determined. Most studies utilize cells obtained from whole blood or visceral organs rather than cutaneous immune cells. This has further hampered our understanding of the contribution of cutaneous cell populations to viral clearance versus systemic spread. The early events that predict viral containment in the skin, vis-à-vis unrestricted replication and dissemination to the blood and subsequently to other organs, remain to be elucidated. A vital question is whether local skin events can portend subsequent disease severity.

### 9.2. Do Tick Life Stage and Species Play a Role in Transmission/Severity?

Generally, tick larvae and nymphs infest small mammals and birds, while adults prefer larger mammals, including humans. While larvae are unlikely to infest humans, transovarial transmission is possible, making larvae potential vectors of illness. The larval stage is extremely small and may often go unnoticed on a human host, thereby preventing documentation of larval infestation. Whether larvae can penetrate deep enough into the skin and whether the feeding duration is long enough to transmit CCHFV is unknown.

### 9.3. What Is the Effect of Tick Saliva on Transmission?

Tick saliva contains immunomodulatory compounds that are known to enhance virus entry and impede immune cell activation and trafficking. However, for CCHFV, the exact molecules involved in these processes are unknown, understudied, or have been extrapolated from studies on other tick-borne viruses. Furthermore, functional studies that explore how these salivary factors alter precise pathways in skin cells are largely still lacking. This includes how tick saliva aids viral transmission and alters type I and III IFN pathways, the complement cascade, inflammasome activation, and cytokine pathways. Following attachment and feeding, ticks modify the cutaneous environment. It has not yet been established how long these changes last, and the effects that they may have on local immunity remain to be investigated.

### 9.4. Can New Approach Methodologies Unlock New Insights?

Most mechanistic studies are performed in mice, where the skin differs immunologically and structurally from that of humans. To mimic virus transmission during tick feeding, ticks are secured in non-physiological sites, which further impacts translatability. There is a lack of standardized ex vivo human-based approaches, such as biopsies, explants, and organoids, which could contribute much-needed insight into CCHFV kinetics, spatial spread, and immune cell interactions in the skin in its native architecture. Incorporating live tick feeding into such systems would fundamentally improve our knowledge of CCHFV transmission dynamics.

### 9.5. What Is the Contribution of Local Immune Memory?

Immune memory protects the host from disease upon secondary exposure to the same pathogen. There are few systematic studies that investigate the frequency of CCHFV reinfection. The first reported instance of CCHFV reinfection in an endemic area occurred in 2023 [302]. It was previously thought that CCHF infection confers long-lasting protection, but there are no longitudinal studies examining the durability of the immune response. Additionally, the immune correlates of protection have not been defined. Immune memory in the skin was long thought to be mediated by adaptive immune cells only, but there is increasing evidence that both innate immune and non-immune cells, such as macrophages and epidermal stem cells, respectively, undergo genetic and metabolic reprogramming leading to the formation of non-specific immune memory [303,304]. What role does cutaneous immune memory play in preventing CCHFV infection? Does this memory exacerbate or mitigate cutaneous and systemic manifestations of the disease upon secondary exposure?

### 9.6. Do Early Immune Cell Dynamics Shape Cutaneous Manifestations of CCHFV?

CCHF, like other hemorrhagic fevers, is characterized by coagulopathies and a dysregulated immune response defined by the aberrant production of cytokines by infected and bystander cells. Another feature of CCHF is the development of a spectrum of cutaneous hemorrhagic lesions that reflect injury to the infected dermal endothelium. There are no studies on early skin lesions to define which cells are activated at different stages of the disease and how these differ between mild and severe cases. Tregs are known to limit virus-induced immunopathology. Given that CCHF is characterized by an overactive immune response in severe cases, it would be interesting to evaluate the contribution of T_reg_ cells to immune regulation and their impact on disease control. Determining the role of various cutaneous cells in shaping the local immune response and viral control versus inducing immunopathology would provide valuable insight that could lead to the development of effective countermeasures.

## 10. Conclusions

The contribution of the skin to the control of tick-borne viruses is understudied and often overlooked as a critical site for early amplification and dissemination of tick-borne pathogens. The trajectory of illness may be defined by the early, intricate dynamics at the tick–host–virus interface. As the etiological agent of a severe and often fatal illness, CCHFV pathogenesis in human skin is an important area of research. The integration of novel approaches and models will serve to increase our understanding of the cutaneous response to tick-borne transmission of CCHFV. Overall, viewing the skin as a critical organ involved in CCHFV pathogenesis offers a unique perspective for controlling and preventing transmission and disease.

## Figures and Tables

**Table 1:** *Hyalomma* spp. Salivary Molecules and Their Putative Effector Functions on the Cutaneous Immune Response.

Tick Species	Salivary Molecule	Known/Putative Function	Relevance to Skin Immunity	Reference
*Hyalomma anatolicum*	Antigen I–III	Immediate and delayed hypersensitivity reactions in sensitized rabbits	Elicits local immune memory and inflammation; potential modulation of skin-resident T cells	[38]
	HAMpin-1 (serine protease inhibitor)	Complement system modulation	Inhibits local complement-mediated inflammation and opsonization in skin	[39]
	–	Inhibition of bovine platelet aggregation	Limits clotting at bite site; reduces platelet-driven inflammatory signaling	[40]
	–	Anti-inflammatory activity	Direct suppression of innate immune responses in the skin	[41]
	–	High antioxidant activity; GSH depletion in females	Detoxifies reactive oxygen species (ROS); weakens oxidative burst by skin phagocytes	[42]
	Time-based secretion profiles	Dynamic expression of proteins over time during feeding	Timed suppression or redirection of skin immune responses across feeding stages	[43]
*Hyalomma asiaticum*	BIF (B-cell inhibitory factor)	Inhibits B-cell proliferation	May suppress local humoral responses in skin-draining lymph nodes	[44]
	Hyalomin-A & Hyalomin-B	Suppress cytokine secretion; ROS detoxification	Suppresses proinflammatory cytokines and reduces oxidative microenvironment	[45]
	Ha24	Lipocalin with specific histamine binding activity	Reduce host inflammatory response	[46]
*Hyalomma dromedarii*	Dromaserpin	Anti-hemostatic activity (serpin family)	Reduces clot-driven immune activation; promotes immunosuppressive feeding niche	[47]
	P5	Thrombin inhibitor	May suppress clotting and thrombin-mediated inflammation, dampening skin immune activation	[48]
	SGE	Anti-proliferative, anti-viability, apoptotic effects of SGE	May directly damage or suppress skin immune cell populations (e.g., Langerhans or dermal DCs)	[49]
	–	Gender differences in secreted proteins	Possible sex-specific modulation of skin immunity	[50]
	–	Proteome analysis available	Resource for identifying novel modulators of skin immunity	[51]
*Hyalomma excavatum*	SGE	Antiangiogenic effects of SGE	Reduces neovascularization; may impair immune cell recruitment to bite site	[52]
	–	Prostaglandin synthesis (PGE_2_, PGF)	Modulates inflammation and vasodilation in the skin microenvironment	[53]
	–	Full sialome available	Enables systems-level analysis of immunomodulatory proteins	[54]
*Hyalomma marginatum*	SGE	Inhibits APC migration	Prevents DC trafficking to lymph nodes; hinders initiation of adaptive immune responses	[33]
*Hyalomma rufipes*	Hyalomin-1	Thrombin inhibitor	Anti-coagulant; reduces inflammation driven by thrombin signaling	[55]

“SGE”: salivary gland extract; “–”: no specific salivary molecule.

## Data Availability

No new data were created or analyzed in this study.

## Abbreviations

α	alpha
β	beta
θ	theta
AFA	Antimicrobial fatty acids
AMP	Antimicrobial peptides
APC	Antigen-presenting cell
ATR	Acquired tick resistance
BIF	B-cell inhibitory factor
CCHFV	Crimean-Congo hemorrhagic fever virus
CD206	Mannose receptor
CD207	Langerin
cDC	Conventional dendritic cells
cDC2	Type 2 conventional DC
CHIKV	Chikungunya virus
CLR	C-type lectin
CLA	Cutaneous lymphocyte-associated antigen
DAMP	Damage-associated molecular patterns
DC	Dendritic cell
dDC	Dermal dendritic cell
DENV	Dengue virus
DETC	Dendritic epithelial T cell
DUGV	Dugbe virus
EBOV	Ebola virus
ECM	Extracellular matrix
FAS	FS7-associated cell surface antigen
FceR1	High-affinity IgE receptors
GM-CSF	Granulocyte-monocyte colony-stimulating factor
HA	Hemagglutinin
hCAMP	Human cationic antimicrobial peptide
HIV	Human Immunodeficiency Virus
HIF	Hypoxia inducible factor
HPV	Human Papillomavirus
HSC	Hematopoietic stem cell
HSV	Herpes simplex virus
IAV	Influenza A virus
ICAM-1	Intercellular adhesion molecule 1
IL	Interleukin
ILCs	Innate lymphoid cells
iSALT	Inducible skin-associated lymphoid tissue
IFN	Interferon
iNOS	Nitric oxide synthase
IRF	Interferon regulatory factors
LC	Langerhans cell
LDLR	Low-density lipoprotein receptor
LLEC	Long-lived effector cells
LPS	Lipopolysaccharide
LTi	Lymphoid tissue inducer
Mφ	Macrophage
MAIT	Mucosal-associated invariant T cell
MAPK	Mitogen-activated protein kinase
MAYV	Mayaro virus
MCP-1	Monocyte chemoattractant protein-1
MDA5	Melanoderma Differentiation-Associated gene 5
MHC	Major histocompatibility complex
MPEC	Memory precursor effector cell
MPS	Mononuclear phagocyte system
moDC	Monocyte-derived DC
NET	Neutrophil extracellular trap
NHP	Non-human primates
NF-kB	Nuclear factor- kB
NK	Natural killer cells
NKG2D	Natural killer cell receptor
NLR	Nod-like receptors
NO	Nitric oxide
PAMP	Pathogen-associated molecular pattern
PBMC	Peripheral blood mononuclear cell
pDC	Plasmacytoid dendritic cells
PI3K	Phosphatidylinositol 3-kinase
POWV	Powassan virus
PRR	Pattern recognition receptor
RIG-I	Retinoic Acid-Inducible gene
RLR	Rig-I-like receptors
ROS	Reactive oxygen species
RSV	Respiratory syncytial virus
SAT	Saliva-assisted transmission
SALT	skin-associated lymphoid tissue
SFV	Semliki Forest virus
SGE	Salivary gland extract
SLECs	Short-lived effector cells
SLNs	Skin-draining lymph nodes
SLO	Secondary lymphoid organs
STAT1	Signal Transducer and Activator of Transcription 1
TBEV	Tick-borne encephalitis virus
TCR	T cell receptor
TGF	Transforming growth factor
T_FH_	T follicular helper
T_H_	T helper
TLR	Toll-like receptors
TNF	Tumor necrosis factor
T_reg_	Regulatory T cell
T_RM_	Resident memory T cell
TOSV	Toscana virus
TRAIL	Tumor necrosis factor-related apoptosis-inducing ligand
USUV	Usutu virus
VACV	Vaccinia virus
VEGF	Vascular endothelial growth factor
WNV	West-Nile virus
ZIKV	Zika Virus
